# Malignancies and glomerulonephritis: when to suspect and when to screen?

**DOI:** 10.1093/ckj/sfaf101

**Published:** 2025-04-10

**Authors:** Ahmet Murt, Ilay Berke, Annette Bruchfeld, Fernando Caravaca-Fontán, Jürgen Floege, Eleni Frangou, Safak Mirioglu, Sarah M Moran, Stefanie Steiger, Kate I Stevens, Onno Y K Teng, Andreas Kronbichler

**Affiliations:** Department of Nephrology Clinic, Istanbul University-Cerrahpasa, Cerrahpasa Medical Faculty, Istanbul Turkey; Department of Immunology, Aziz Sancar Institute of Experimental Medicine, Istanbul University, Istanbul, Turkey; Department of Internal Medicine IV, Nephrology and Hypertension, Medical University Innsbruck, Innsbruck, Austria; Department of Health, Medicine and Caring Sciences, Linköping University, Linköping, Sweden; Department of Renal Medicine, Karolinska University Hospital and CLINTEC Karolinska Institutet, Stockholm, Sweden; Instituto de Investigación Hospital 12 de Octubre (imas12), Madrid, Spain; Division of Nephrology, Rheinisch-Westfälische Technische Hochschule (RWTH) Aachen University Hospital, Aachen, Germany; Department of Nephrology, Limassol General Hospital, Limassol, Cyprus; University of Nicosia Medical School, Nicosia, Cyprus; National and Kapodistrian University of Athens Medical School, Athens, Greece; Department of Immunology, Aziz Sancar Institute of Experimental Medicine, Istanbul University, Istanbul, Turkey; Division of Nephrology, Bezmialem Vakif University Hospital, Istanbul, Turkey; Division of Nephrology, Department of Internal Medicine, Istanbul Faculty of Medicine, Istanbul University, Istanbul, Turkey; Cork University Hospital, University College Cork, Cork, Ireland; Division of Nephrology, Department of Medicine IV, Ludwig-Maximilians-University Hospital Munich, Munich, Germany; Glasgow Renal and Transplant Unit, Queen Elizabeth University Hospital, Glasgow, UK; Center of Expertise for Lupus, Vasculitis and Complement-mediated Systemic disease (LuVaCs), Department of Nephrology, Leiden University Medical Center, Leiden, the Netherlands; Department of Internal Medicine IV, Nephrology and Hypertension, Medical University Innsbruck, Innsbruck, Austria; Department of Health, Medicine and Caring Sciences, Linköping University, Linköping, Sweden

**Keywords:** cancer, glomerulonephritis, malignancy, membranous nephropathy, paraneoplastic syndrome

## Abstract

Glomerular diseases may occur secondary to malignancies. Age-specific cancer screening is recommended for patients with glomerular diseases and may be extended based on the specific risk associated with the detected histopathologic pattern. Membranous nephropathy is the prototype of cancer-associated glomerulonephritis, with 10% of cases presenting with malignancy within a year from diagnosis. Among antigens that are expressed in patients with membranous nephropathy thrombospondin type 1 domain-containing 7A and neural epidermal growth factor-like-1 are often reported in patients with underlying malignancies. However, the risk of having a concurrent malignancy does not exceed 25%–30% when these antigens are expressed. While less frequent in other glomerulonephritides, co-occurrence of malignancy is reported in a substantial proportion of glomerular diseases including IgA nephropathy, podocytopathies with prominent podocyte foot process effacement such as minimal change disease as glomerular lesion pattern, amyloidosis, C3 glomerulopathy, monoclonal immunoglobulin deposition disease, or immune-complex-mediated glomerulonephritis. Treatment of malignancy-associated glomerulonephritis is usually directed toward treatment of the underlying malignancy with combinations of surgery, chemotherapy, and/or radiotherapy. Moreover, relapse of the malignancy may result in recurrence of glomerulonephritis. Refractoriness of glomerulonephritis to initial therapy may be due to an occult primary malignancy that was not diagnosed during initial cancer screening. In such a scenario a step-up diagnostic approach is recommended. In addition, re-screening may be sensible for relapsing patients who carry higher risks for cancer including patients of older age and those with a smoking history. This review focuses on the description of malignancies in the context of glomerular diseases and provides practical guidance on screening.

## Graphical Abstract

**Graphical Abstract:**
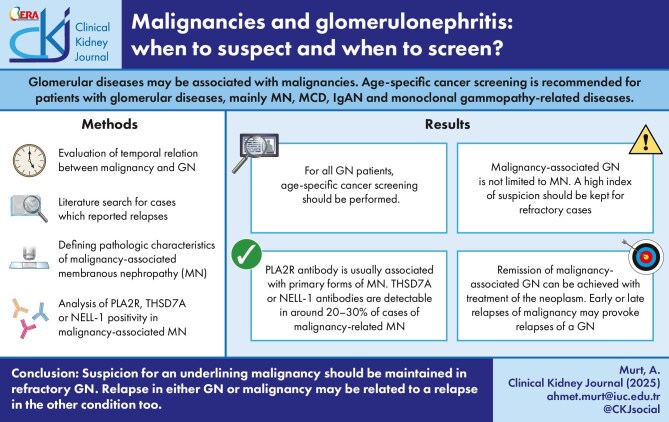

EXECUTIVE SUMMARY

- Glomerulonephritis (GN) may occur secondary to an underlying malignancy (also referred to as paraneoplastic GN). GN and malignancy may also incidentally co-occur. For all patients with GN, age-specific cancer screening should be performed and should be individually adjusted according to risk factors for malignancy. Emphasis should be placed on encouraging participation in any existing national screening programs that may exist in individual countries.
- Malignancies at the time of GN diagnosis may be occult. Such GN cases are typically treated as if these were primary GN. However, a high index of suspicion should be maintained in patients with refractory disease or in the setting or frequent relapses. More stringent cancer screening, including the use of more sensitive imaging studies should be performed in such cases. Occult malignancies generally manifest within 1 year of initial GN diagnosis.
- Membranous nephropathy (MN) is the prototype of paraneoplastic GN; the presence of phospholipase A2 receptor (PLA2R) antibody is usually associated with primary forms of MN. Thrombosponding type 1 domain-containing 7A (THSD7A) or neural epidermal growth factor-like 1 protein (NELL-1) antibodies are detectable in ∼20%–30% of cases of malignancy-related MN.
- Paraneoplastic GNs are not limited to MN and can be associated with all glomerular lesions.
- Remission of malignancy-associated GN can be achieved with treatment of the neoplasm. Early or late relapses of malignancy may provoke relapses of a GN, initially related to that malignancy. Relapsing GN may also be one of the first signs of cancer recurrence.

## INTRODUCTION

Glomerular disease may occur secondary to other causes including autoimmune disease, infection, drugs, or malignancy. Malignancy-related glomerular disease should be suspected particularly when observed in close temporal relation to the detection of cancer. The clinical practice guideline of the Kidney Disease: Improving Global Outcomes for the management of glomerular disease underlines the association of membranous nephropathy (MN), IgA nephropathy (IgAN), immune-complex-mediated glomerulonephritis, podocytopathies with prominent podocyte foot process effacement, often referred to as minimal change disease (MCD), and anti-neutrophil cytoplasmic antibody (ANCA)-associated vasculitis (AAV) with malignancies [1]. The literature also includes a wider variety of glomerular lesions observed concomitantly with a malignancy. These reported associations underscore the necessity of appropriate age-specific cancer screening in patients with GN [2]. Tumors may be detected either at initial presentation or later in the disease course, especially in patients with refractory or relapsing glomerular disease [3]. Some tumors are initially occult, a situation where malignancy could not be diagnosed by initial screening and cancer diagnosis may be delayed. A high index of suspicion should always be maintained in patients with risk factors for malignancies including the type of glomerular disease (e.g., MN), older age at time of presentation, and exposure to carcinogens [4].

This review aims to provide a comprehensive framework for cancer screening in patients with GN, with a focus on exploring the temporal relationships—both early and late—between malignancy relapse and the recurrence of GN. The clinical implications of GN recurrence in relapsing malignancies are also discussed. Understanding the relationship between malignancies and glomerular diseases will enable better management of malignancy-related GN as well as timely diagnosis of some malignancies.

### Malignancy-associated glomerulonephritis and cancer screening

Some of the glomerular disease patterns/lesions are more likely to be associated with malignancy. Most of the evidence gathered over recent decades links these lesions with MN, MCD, IgAN, and membranoproliferative GN (MPGN) [4–6]. Less evidence exists for other GN forms, however, incidental cases with concomitant malignancies have been reported for many other types of glomerular disease.

**Figure 1:**
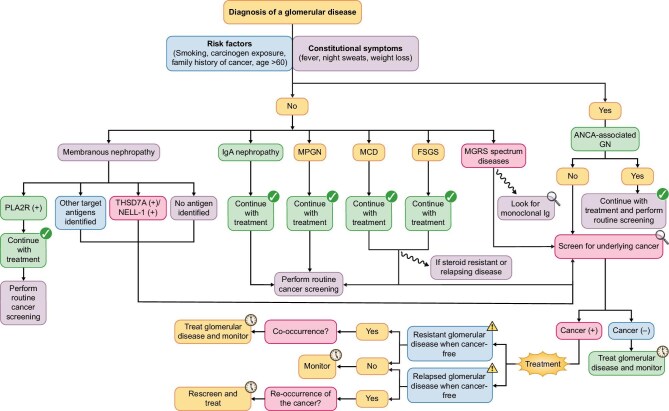
A proposed algorithm for cancer screening, surveillance for underlying or concomitant malignancies, and treatment of glomerular diseases. Screening for underlying malignancy should be based on individual risk factors and presenting features of the patients, i.e. the presence of constitutional symptoms. Some glomerular diseases tend to occur more frequent as a paraneoplastic syndrome, especially patients with MN or those within the monoclonal gammopathy of renal significance spectrum. Constitutional symptoms are common for severe systemic autoimmunity and should not trigger intensified screening; only if the symptoms are persisting or part of a renal-limited disease such as a podocytopathy. If patients are resistant to treatment measures or relapse early in the disease course, consider a more intensified search for occult malignancies.

Cancer screening is recommended for patients with certain glomerular lesions [2]. Especially in patients with MN, age-specific cancer screening is crucial. A proposed algorithm for the target population who should be screened and kept under surveillance for underlying or concomitant malignancies is described in Fig. 1. Basic imaging techniques, such as a chest X-ray and an ultrasound of the abdomen are broadly available and help excluding a broad range of malignancies. When high suspicion of an underlying malignancy is present, the exclusion of common malignancies, such as skin or urogenital tract cancer is warranted and should be performed. When inflammation and B symptoms such as fever, night sweats, and weight loss are present and not explained by glomerular disease, F-fluorodeoxyglucose (FDG)-positron emission tomography (PET)/computed tomography (CT) is a logical diagnostic step-up because it can detect metabolically active sites of malignancies [7]. A diagnostic strategy of cancer screening in these patients is provided in Table 1.

**Table 1:** A proposed screening strategy for patients with glomerular diseases and suspected underlying malignancy.

History, signs/symptoms	Ask for constitutional symptoms; family history; thorough physical examination, especially for palpable lymph nodes, hepatomegaly, or splenomegaly; ask for symptoms of prostate enlargement
Laboratory tests	Complete blood count, lactate dehydrogenase, liver and kidney function tests
Viral serology	Hepatitis B and C, human immunodeficiency virus, EBV, human papillomavirus
Tumor markers^a^	Carcinoembryonic Antigen, CA 125, CA 19–9, CA 15–3, Alpha Fetoprotein, Prostate-specific Antigen
Initial imaging	Chest X-ray, abdominal ultrasound
Step-up imaging techniques upon indication	CT of the chest, abdomen, and pelvis; consider PET/CT scan when there are signs of unexplainable inflammation
Routine age-specific screening	Fecal occult blood test, PAP smear, mammography, gastroscopy, colonoscopy
More specific tests	Urine cytology or cystoscopy for patients with risk factors and ongoing and unexplainable hematuria (especially when exposed to alkylating agents) Bone marrow biopsy when hematologic malignancy is suspected

a Consider the impact of nephrotic syndrome and/or chronic kidney disease on serum levels.

Abbreviation: CT, computed tomography; EBV, Epstein-Barr virus; PET, positron emission tomography.

Glomerular diseases associated with malignancies are considered part of the spectrum of paraneoplastic syndromes. Paraneoplastic GN should be suspected when the glomerular disease and malignancy are diagnosed simultaneously and when there are no evident triggering events for GN [5]. Generally, complete remission of the underlying glomerular disease should be achieved after remission of the malignancy by chemo/radiotherapy, complete surgical removal of the tumor, or both [4]. Paraneoplastic GN has a tendency to recur with recurring malignancy.

In MN, different antigens have been identified which may stratify patients and their risk of underlying malignancy. Among them only antibodies against the phospholipase A2 receptor (PLA2R) and the PLA2R antigen detection by immunofluorescence are broadly available. Presence thereof is usually associated with primary MN and thus confer a high predictive value to rule out underlying malignancy.

Other antigens are only detectable in specialist laboratories but most of them have an association with a trigger/offending agent. The second antigen to be detected in MN, was thrombospondin type 1 domain-containing 7A (THSD7A). Expression of THSD7A on some malignant cells makes is it a noteworthy antigen within the framework of malignancy-associated MN [6]. A meta-analysis showed that 6% to 25% of THSD7A positive MN cases had concurrent malignancies [8]. Another recently discovered antigen, neural epidermal growth factor-like-1 (NELL-1) is likewise linked to malignancies with 10% to 30% of NELL-1 positive MN cases associated with concurrent malignancy [9, 10]. Using laser microdissection of the glomeruli together with mass spectrometry other novel antigens have been identified in MN [11] including Protocadherin 7, present in 2% of MN cases, and which has a 20% probability of being associated with malignancy [12].

According to the Mayo Clinic consensus report, THSD7A and NELL-1 are the main MN antigens which should arouse suspicions of an underlying malignancy [13]. Table 2 summarizes the percentages of selected MN antigens in patients with primary MN and malignancy-related MN.

**Table 2:** The rate of malignancy as reported in studies with proposed antigens implicated in MN.

Antigen	Rate of positivity in primary membranous nephropathy	Approximate incidence of MN antigens [13]	Rate of underlying malignancy when reported positive
PLA2R [13, 34]	70%–80%	55%	Up to 5%^a^
THSD7A [8, 34]	3%	2%	6%–25%
NELL-1 [9, 10]	1%–2%	10%	10%–30%
PCDH7 [12]	2%	2%	20%

a Mainly reported in investigations from China (even higher frequencies); rarely reported/seen in Caucasian populations.

Abbreviation: PCDH7, Protocadherin 7; PLA2R, phospholipase A2 receptor; THSD7A, thrombospondin type-1 domain-containing 7A; NELL-1, neural epidermal growth factor-like 1.

IgAN, which is related to mucosal immune dysregulation might be observed in relation to mucosal tumors. Although the exact mechanism remains to be defined, aberrant glycosylation patterns of transformed cancerous cells may result in high amounts of galactose-deficient IgA1 (Gd-IgA1) and generation of autoantibodies against Gd-IgA1 [14]. The reported malignancies in IgAN often originate from the buccal cavity, nasopharynx, and respiratory tract suggesting a link between ongoing mucosal immune dysregulation and cancer [15]. Renal cell carcinoma (RCC) is also reported to be associated with IgAN. For patients >50 years of age, a diagnosis of IgAN might coincide with presence of RCC, likely influenced by paraneoplastic deposition of mesangial IgA [16].

MCD is known to be associated with Hodgkin's disease and other lymphomas [17]. The underlying pathophysiological mechanism for lymphocytic proliferation is not well established but it may include viral involvement [18]. In a retrospective French pediatric series of 11 nephrotic syndrome patients, Epstein–Barr virus (EBV) was found in five cases and Human Herpesvirus-8 in two [19]. Particularly in the setting of steroid-resistant or relapsing MCD, Hodgkin's disease should be considered as a potential underlying risk factor and a detailed lymph node examination should be performed [20]. Specifically, Hodgkin's disease needs to be excluded in patients with nephrotic syndrome and systemic symptoms (referred to as constitutional symptoms), such as fever, night sweats, and weight loss. Of note, a recent systematic literature review identified 86 cases with MCD and solid organ malignancies. Of these, malignant thymoma (34.9%), kidney (14.0%), lung (12.8%), and gastrointestinal (12.8%) tumors were the leading malignancies. An almost equal split in occurrence was reported, as in 40.7% the neoplasm preceded onset of MCD, 27.9% had a co-occurrence of both entities and in 31.4% the tumor diagnosis followed the diagnosis of MCD by an average of 12 months. These patients also had an unexpected high frequency of kidney replacement therapy requirement [21]. Of note, an MCD pattern on kidney biopsy as part of an autoimmune podocytopathy does not typically cause constitutional symptoms, while this is expected in the case of solid malignancies.

Multiple myeloma may cause glomerular diseases, including light chain (AL) amyloidosis, C3 glomerulopathy, monoclonal immunoglobulin deposition disease, proliferative GN with monoclonal IgG deposits, which is either monoclonal gammopathy of renal significance (90%) or malignancy-related (10%) [22], fibrillary, or immunotactoid GN [23]. The identification of such entities on kidney histopathology should guide nephrologists to search for monoclonal gammopathies.

Other types of glomerular diseases are likely associated with malignancies: however, accurate estimation of their frequencies is challenging. One explanation is that a per protocol cancer screening is performed in certain types of GN, such as MN, while a per indication cancer screening is performed in others thus leading to a lower number of cancer diagnosis. Another entity often presenting with tumor-like masses is IgG4-related disease, where different organ manifestations can be detected [24]. The disease can affect the kidney and manifests most commonly as tubulointerstitial nephritis or MN, and kidney disease was a frequent manifestation in the recently reported phase 3 trial testing efficacy of inebilizumab [25], and IgG4-related kidney disease frequently leads to acute kidney injury [26]. In addition, AAV can present with large masses; however, granulomatous masses in the kidney are rarely reported [27]. Additionally, malignancies diagnosed during the longer follow-up may complicate the cause-and-effect relationship, as many immunosuppressive drugs used to treat glomerular diseases are linked with oncogenicity.

### Relapsed glomerulonephritis: do we need to re-screen for malignancy?

During the initial presentation of glomerular disease, cancer screening may not always reveal an underlying malignancy and in many cases, immunosuppression is thus prescribed to manage what is believed to be a primary GN. Subsequently, malignancies may be recognized during follow-up. There is generally a diagnostic delay of ∼6–12 months after initial GN diagnosis in those patients [28]. A high degree of suspicion for malignancy is imperative when the underlying glomerular disease is refractory to therapy (Fig. 1).

MN represents the prototype of malignancy-related GN. Therefore, most algorithms describing the approach of malignancies in GN are derived from MN cases. Recently, among 42 documented malignancy-associated MN patients, only 14 malignancies were identified before the diagnosis of MN [29]. The median time to detect malignancies after MN diagnosis was 14 months (interquartile range 4–18 months). Immunosuppressive therapy was initiated in 11 out of 13 patients who had negative initial cancer screening. Remission could not be achieved in >50% of these cases. In this study, 6 of 10 patients who attained cancer remission also achieved remission of MN, whereas those who did not reach cancer remission also did not achieve remission of MN. Patients with malignancy-associated MN were older and less likely to have positive glomerular staining for phospholipase A2 receptor. In a study by Burstein *et al*., in the pre-PLA2R era, malignancies were detected in 10% of the patients who were presumed to have primary MN [30]. In those who had their malignancy diagnosed after MN the delay was 2 to 7 months. Three explanations might have relevance for the delay of cancer diagnosis: malignancies occurred after MN; malignancies were occult at the time of MN diagnosis; or proper malignancy screening was not initially performed for some of patients. In principle, malignancies identified prior to or within the first year of follow-up are assumed to be related to the onset of GN. If malignancy is detected at a later time point, it is more likely that a *de novo* cancer has occurred. Recurrence of disease should trigger further screening for malignancy and, if available, the assessment of antibodies or tissue-specific antigens (even if previously tested and negative) such as those against PLA2R and THSD7A. Such an approach has been taken in the case of a 68-year-old male patient, who initially was THSD7A-negative and was subsequently treated as primary MN. However, the patient presented with a relapse of MN 1 year after the initial diagnosis and kidney biopsy showed THSD7A-positive MN. A bladder tumor was detected [31] and resection of the tumor led to remission of MN, suggesting an association of malignancy with MN.

Clinical presentation of primary and malignancy-associated MN might be similar and histopathology might provide limited clues to discriminate between them [32]. Treatment approach between the two entities is, however, different. While immunosuppressive therapy constitutes the main treatment in primary MN, management of the patient with malignancy-associated MN should start with anti-cancer treatment. Although glomerular PLA2R staining and serologic anti-PLA2R positivity typically point toward primary MN, this does not necessarily exclude all malignancies. Interestingly, in a 26-year-old female patient with RCC-related MN RCC cells stained positive for PLA2R [33]. Serologic analysis from different cohorts showed that up to 5% of the PLA2R-positive MN cases may have concomitant malignancies [13, 29]. In a study from China which involved 36 malignancy-associated MN, 33% of the patients had glomerular PLA2R staining and serum anti-PLA2R antibodies [34]. In another investigation from China, 3 out of 10 malignancy-associated MN patients had positive anti-PLA2R antibodies in serum [35]. PLA2R-positive malignancy-associated MN cases were mostly clustered in studies from China. It should be noted that, for MN in general, PLA2R positivity was reported in 71% in a European cohort, while it was 48% in an US cohort [13]. On the other side, presence of NELL-1 was found in 23% of cases in the discovery cohort from the US, while it was 6% in European validation cohorts and most NELL-1 positive MN cases from European validation cohorts had concomitant malignancies [9]. Also, Chinese MN cases had a lower likelihood of expressing NELL-1 [36]. By contrast, THSD7A positivity has been reported more frequently in Japanese MN patients than in European/American cohorts, being present in ∼9% [37]. These findings demonstrate that antigen expression in different forms of MN may vary according to geographical regions, genetic background, methods used in different institutions or access, suggesting the need for global standardization.

Uniform thickening of the glomerular basement membrane (GBM) is observed in primary MN and subepithelial IgG deposition is mainly of the IgG4 subtype [38]. Of note, no proliferation is usually encountered. On the contrary, in malignancy-associated MN, an uneven distribution of subepithelial deposits and additional subendothelial deposits is often reported. The dominant IgG deposition is of IgG1 and IgG2 [34]. In addition, endocapillary hypercellularity and mesangial proliferation may also be seen. In malignancy-associated MN, the kidney tissue contains more inflammatory cells with hypercellularity in the mesangium and leukocyte infiltration in the glomerular capillary lumen. The proposed cut-off to suspect malignancy is eight inflammatory cells per glomerulus [32]. This was independently confirmed by a study of Hoxha *et al*., who also showed that the presence of more than eight inflammatory cells per glomerulus was associated within malignancy in MN [39]. Such findings should guide clinicians for re-evaluating clinical history of the patients and any discrepancy between the clinic and morphological findings should raise the suspicion for an underlining secondary form of MN. For PLA2R-negative cases, anti-THSD7A positivity may provide additional clues to suspect malignancy-associated MN. In support of this proposition, cancerous tissue of the colon and breast was previously shown to have high levels of THSD7A expression [40]. A prior study showed that 20% of patients with THSD7A-associated MN had malignant tumors that were detected after the diagnosis of MN was made [41]. Together, these demonstrate that re-screening for incident malignancies is warranted in patients with MN particularly in the absence of treatment response and mainly in PLA2R-negative but THSD7A-positive patients. THSD7A was also detected in follicular dendritic cells of lymph nodes [39, 42]. This suggests that malignancies with lymph node involvement have a greater tendency to cause GN. Thus, the presence of lymphadenopathies in any imaging study of GN patients should direct physicians to undertake more detailed cancer screening.

In some MN patients who present with nephrotic syndrome and despite high suspicion for cancer, i.e. in the context of heavy smoking or exposure to carcinogens such as asbestos, the initial evaluation may rule out malignancy. Although immunosuppressive therapy may generate remission in 20%–25% of such cases, tumors may become apparent during follow-up and recurrence of nephrotic syndrome may be observed early in the disease course in malignancy-associated MN [8, 13].

An association between disease relapse and concomitant presence of malignancy has also been reported in glomerular disease with little or no tendency of relapse. In a 37-year-old male patient with anti-GBM disease, anti-GBM antibodies turned negative after treatment with plasma exchange, glucocorticoids, and cyclophosphamide. However, when anti-GBM antibodies recurred 1 year later, the patient was also diagnosed with T cell large granular lymphocytic leukemia (LGL) [43]. As LGL is often indolent, LGL might have been present at initial presentation 1 year previously, but the authors reported that a lack of blood samples hindered further investigations. Further examples are highlighted in Table 3 [31, 44].

**Table 3:** Examples of cases where malignancy was detected at the time of relapse, within a window of 12 months from initial diagnosis.

Cases	Age at diagnosis	Initial diagnosis	Specific findings at diagnosis	Time to relapse	Malignancy detected at the relapse	Specific findings at GN relapse
1 [31]	68	Membranous nephropathy	THSD7A negative	1 year	Bladder cancer	THSD7A positive
2 [43]	37	Anti-GBM disease	Response to plasma exchange, steroids and CYC	1 year	T cell LGL leukemia	Anti-GBM antibodies recurred
3 [44]	68	Immunotactoid glomerulopathy	Remission after prednisolone monotherapy	1 year	DLBCL	Treatment of DLBCL led to remission of nephrotic syndrome

Abbreviations: CYC, cyclophosphamide DLBCL, diffuse large B cell lymphoma; GBM, glomerular basement membrane; GN, glomerulonephritis; LGL, large granular lymphocyte; THSD7A, thrombospondin type-1 domain-containing 7A.

As malignancy-related glomerular diseases have a higher tendency of recurrence than primary GN, nephrologists should be suspicious of the co-existence of malignancies in patients with rapidly and frequently recurring proteinuria. In patients with risk factors for cancer, such as family history, smoking, older age (>65 years), or other carcinogen exposure, cancer screening should be extended and an intensive work-up should be carried out to look for occult malignancies.

Although cancer treatment is the main strategy in malignancy-related MN, in some cases, MN may not remit after resolution of cancer. Additionally, the tumor cannot always be identified during relapses of glomerular diseases. These scenarios may point to coincidental occurrence of the two rather than a causal relationship. Relevant immunosuppressive therapy should be commenced in such cases in particular if PLA2R antibody is concomitantly positive, where coincidental occurrence of MN and malignancy is more likely [45]. In line with this, malignancy-associated MN patients who had PLA2R positivity also had persistent or relapsing proteinuria after tumor resection [35]. Rituximab is the most reasonable option for the treatment of MN patients who have concomitant malignancies and PLA2R positivity [46], as carcinogenesis was not reported as a serious adverse effect of rituximab use in MN [47]. Similarly, in AAV, a secondary GN requiring intensive immunosuppression [48], malignancy risk with rituximab was not found to be increased in comparison to the general population [49]. By contrast, the use of alkylating agents such as cyclophosphamide might be linked to *de novo* or recurrent malignancies [50]. Although a relation between AAV and malignancies is not clear [51], a pauci-immune crescentic GN was reported 16 months after the diagnosis of lung adenocarcinoma in a 66-year-old male patient [52]. Proteinase 3-ANCA was concomitantly positive. Inflammation caused by the tumor was suggested to have contributed to AAV.

Glomerular diseases may also precede malignancy and might serve as an indicator for relapse of malignancies. This is independent of the presence of glomerular disease at initial presentation of the malignancy [53]. However, when glomerular disease becomes apparent during a recurrence at a later time point, the possibility of coincidence should be considered. When available, testing for the expression of specific antigens in the tumoral tissue such as THSD7A or NELL-1 may show the association between malignancy and glomerular disease. Interestingly, as MCD and Hodgkin's disease can occur simultaneously, historically an aggressive chemotherapeutic regimen was used to manage recurrence of nephrotic syndrome without the need for clear evidence for a Hodgkin's relapse [54]. Table 4 presents specific case examples where glomerular disease manifested during malignancy recurrence, such as reported cases of MN, IgAN, AAV, and focal segmental glomerulosclerosis [55–58].

**Table 4:** Examples of patient cases where the glomerular disease occurred at the time of relapse of the respective malignancy.

Case	Age	Malignancy	GN initially	Time to relapse	GN at relapse
1 [53]	62	Hodgkin lymphoma	None	11 years	FSGS
2 [55]	68	Esophageal cancer	None	5 years	MN (NELL-1 positive both in kidney and tumor tissue)
3 [56]	59	Gastric adenocarcinoma	None	16 months	IgAN
4 [57]	69	Ovarian granulosa cell tumor	None	39 years	AAV
5 [58]	78	Non-Hodgkin lymphoma	None	9 months	FSGS

Abbreviation: AAV, ANCA-associated vasculitis; FSGS, focal segmental glomerulosclerosis; GN, glomerulonephritis; IgAN, IgA nephropathy; MN, membranous nephropathy.

Apart from inflammation caused by the cancerous tissue, one other important proposed mechanism for paraneoplastic GN is the generation of autoantibodies toward a tumor antigen. Expression of the antigen resulting in the glomerular lesion is expressed by particular tumor types. Thus, certain GN forms in a patient may be caused by specific tumors and not necessarily by all malignancies. This has been exemplified by reports where a second primary tumor did not result in GN recurrence but GN occurred only during the course of the first diagnosed cancer. As an example, in a patient with glottic cancer and concomitant IgAN, hematuria and proteinuria disappeared after the glottic cancer was cured. Prostate and hepatocellular cancers that appeared later in the same patient did not cause a recurrence of IgAN [59].

Even if the association between malignancy and glomerular lesion is well established, GN may not always resolve after adequate treatment of the tumor. For example, MCD usually responds to steroids. However, with concomitant thymoma, steroid-resistant MCD is reported after thymectomy. This is because T cell dysfunction may not recover after thymectomy. Rituximab is often an effective agent to achieve remission in such cases [60]. It is also important to measure antibodies against nephrin in such a scenario, to highlight whether thymoma and MCD are occurring co-incidentally rather than manifesting in association with each other. In some cases, GN may occur after resection of a tumor without any sign of cancer relapse. This points to lymph node involvement with ongoing expression of certain antigens such as THSD7A [61].

Some case series, albeit with small sample size, indicate that the rate of GN relapse may be increased when they are associated with malignancies. In a case series of pauci-immune necrotizing GN, those that were malignancy related had a relapse rate of >30% compared with ∼15% in those with a primary disease [62].

## CONCLUSION

Glomerular lesions may develop secondary to malignancies. Although MN, IgAN, MCD, MPGN, focal segmental glomerulosclerosis, and AAV are the most common types of malignancy-associated glomerular disease, case reports show that a wider range of glomerular disease can be related to malignancies. Age-specific cancer screening is warranted for all patients with glomerular disease including attention to individual risk profiles. Initial screening may not reveal the presence of malignancy and thus there should be a high level of suspicion for treatment-resistant or frequently recurring GN, especially in the first year following GN diagnosis. In such cases, a more detailed cancer re-screening plan may be warranted, incorporating advanced imaging studies such as FDG/PET-CT to evaluate otherwise unexplained inflammation and/or constitutional symptoms. Importantly it should always be borne in mind that a relapse in either GN or malignancy in a patient in prior clinical remission of both malignancy and GN may be related to a relapse in the other condition too.

## Data Availability

No new data were generated or analyzed in support of this research.
